# From Nonalcoholic Steatohepatits to Steatotic Liver Disease: A Long Way

**DOI:** 10.3390/diagnostics13193104

**Published:** 2023-09-30

**Authors:** Helma Pinchemel Cotrim

**Affiliations:** Bahia Medicine School, Federal University of Bahia, Salvador 40026-010, Brazil; helmacotrim@gmail.com

In 1980, Ludwig et al. [1] proposed the term “nonalcoholic steatohepatitis” (NASH) for a histological condition resembling alcohol-related liver disease. This condition was observed in liver biopsy samples from patients with no significant history of alcohol consumption. Within this case series, NASH was noted to be more frequent among women in their fifth decade with obesity and type 2 diabetes mellitus (T2DM). These observations led the authors to suggest that NASH might signify a distinct disease in this population. This study marked a significant milestone in the knowledge of fatty liver disease.

In 1986, Schaffner and Thaler [2] introduced the term “nonalcoholic fatty liver disease” (NAFLD) encompassing a histological broad spectrum that included steatosis, steatohepatitis (NASH), which can progress to cirrhosis, and even hepatocellular carcinoma. NAFLD, currently recognized as the most prevalent liver disease, has reached epidemic proportions, and is acknowledged as a global public health concern. Its most frequent risk factors include cardiometabolic factors such as obesity, T2DM, dyslipidemia and arterial hypertension. Therefore, the NAFLD nomenclature has been subjected to criticism over a considerable period, prompting the introduction of alternative terminologies.

In 2002, during an AASLD-sponsored Single Topic Conference on NAFLD, various aspects of the disease were discussed, and the term “metabolic steatohepatitis” (MESH) was proposed and debated; however, it was not uniformly accepted [3]. Over the past two decades, other suggestions for new nomenclature for NAFLD have emerged [4]. However, a more extensive discussion on the topic arose with the publication of “The International Consensus Panel” involving experts from twenty-two countries [5]. Their aim was to propose the term “metabolic-associated fatty liver disease” (MAFLD) as an alternative for NAFLD. This proposed criterion included the presence of steatosis, associated with one of three clinical conditions: overweight/obesity, T2DM or other metabolic alterations. However, it also permitted considering the association with other liver diseases, including alcohol consumption by the patients. The MAFLD denomination produced discussions and divergent expert opinions within the global community focused on liver diseases. These debates involved viewpoints both in favor of and against changing the name of this hepatic disease. One of the most critical arguments to include alcohol as a factor associated with MAFLD denomination is the absence of the precise definition for the specific level of alcohol consumption.

Alcohol-related liver disease (ALD) holds epidemiological, clinical, and prognostic relevance and it also is considered a public health problem. It also encompasses a wide spectrum, potential progression, and a high morbidity and mortality. Thus, regarding ALD as just a disease associated with MAFLD could underestimate the significance of this form of liver pathology and lead to its inappropriate management. Moreover, despite cardiometabolic conditions constituting the primary risk factors for NAFLD, it also is recognized to be associated with various other causes such as medications, environmental factors, and genetic disorders.

In an article titled “From nonalcoholic fatty liver disease to metabolic-associated fatty liver disease: Big wave or ripple?”, Kang SH et al. [6] suggested that placing emphasis on metabolic dysfunction (MAFLD) could potentially underestimate the prognostic value of hepatic steatosis itself. They emphasized that individuals with non-metabolic NAFLD, excluded based on the MAFLD criteria, might not be thoroughly investigated, and their potential risk might not be adequately assessed. They highlighted that these approaches could influence the prognosis of such patients. In another relevant article, Younossi ZM et al. [7] discussed the proposed name change from NAFLD to MAFLD and emphasized that while NAFLD does not fully reflect the more metabolic risk factors that are more common for this liver disease, the term MAFLD might not be the most appropriate choice.

The authors also suggested the establishment of an international consensus group involving relevant scientific societies, patient advocacy organizations, the bio-pharmaceutical industry, regulatory agencies, and policy makers. This consensus group, through a collaborative meeting, can assess the implications and outcomes of the terminology modification using existing evidence and offer guidance to propel the field forward.

Over the last three years, a long discussion has taken place regarding the new denomination for NAFLD. In June 2023, a significant development occurred with the publication of the “A multi-society Delphi consensus statement on new fatty liver disease nomenclature” [8]. This statement was a collaborative effort involving three major liver disease associations (AASLD, EASL, ALEH) and engaged a diverse panel of 225 experts from 56 countries. The panel also included representatives from patient advocacy organizations, the bio-pharmaceutical industry, regulatory agencies, and policy makers.

An independent committee of experts not directly involved in the nomenclature process provided the final recommendation on the acronym and its diagnostic criteria. Consensus was defined a priori as a supermajority (67%) of votes.

Response rates in the four survey rounds were 87%, 83%, 83%, and 78%, respectively. Among the respondents, 74% considered the NAFLD, a current nomenclature, to be insufficient and supported a name change. The terms “non-alcoholic” and “fatty” were judged stigmatizing by 61% and 66% of respondents, respectively.

The designation “Steatotic liver disease” (SLD) was chosen as a comprehensive term to encompass diverse etiologies associated with steatosis. The term “steatohepatitis” preserved its significance as an important pathophysiological concept. Figure 1 shows the Steatotic Liver Disease (SLD) and the sub-classification.

In summary, SLD has emerged as the novel nomenclature for NAFLD after years of discussion and deliberations. It now presents itself an appropriate terminology for this liver condition. The next focus should be the prioritization of the development of programs to prevent, diagnose, and to treat this liver disease, which is already considered a relevant public health. With its general prevalence across the globe, Steatotic Liver Disease is naturally linked to levels of morbidity and mortality, underscoring its critical impact.

## Figures and Tables

**Figure 1:**
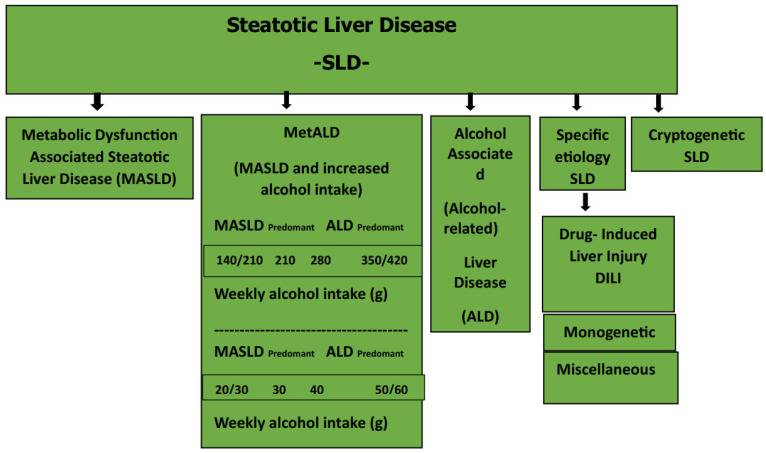
Steatotic liver disease sub-classification [8]. Metabolic-associated steatotic liver disease (MASLD): the presence of at least one of the five cardiometabolic risk factors; alcohol-related SLD: SLD-ALD: alcohol consumption (140–350 g/week/men; 210–420 g/week/women); specific etiology SLD: DILI; monogenetic diseases, miscellaneous; cryptogenic SLD: unknown causes.
